# Side Biases in Euro Banknotes Recognition: The Horizontal Mapping of Monetary Value

**DOI:** 10.3389/fpsyg.2018.02293

**Published:** 2018-11-21

**Authors:** Felice Giuliani, Valerio Manippa, Alfredo Brancucci, Luca Tommasi, Davide Pietroni

**Affiliations:** ^1^Laboratory of Behavioral Economics, Human Center Design and Healthcare, D’Annunzio University of Chieti–Pescara, Chieti, Italy; ^2^Department of Psychological, Health and Territorial Sciences, D’Annunzio University of Chieti–Pescara, Chieti, Italy; ^3^Department of Neuroscience, Imaging and Clinical Sciences, D’Annunzio University of Chieti–Pescara, Chieti, Italy

**Keywords:** banknotes perception, SNARC effect, valence specific hypothesis, body-specific hypothesis, laterality

## Abstract

Money is a special stimulus for humans, because of its relevance in everyday life. However, the basic mechanisms underlying money representation have not yet been fully investigated. Left-right asymmetries in the visual perception and evaluation of monetary value offer such a possibility. The pattern of these asymmetries can contribute to disentangle between numerical and emotional processes possibly involved in banknotes perception. In the present experiment, we tested the recognition of 5€and 100€ banknotes presented in the left and right visual fields. Results show that the 100€ banknote is recognized faster than the 5€ banknote in the Right Visual Field (RVF), while there is no difference in the Left Visual Field (LVF). Our interpretation is that this effect is due to the matching between the positive valence conveyed by the 100€ banknote and the side in which it is mapped (right-positive). We consider this result as evidence of a valence-based recognition of banknotes.

## Introduction

Our brain tends to map quantities and affective valence in relation to individuals’ horizontal space. Specifically, the representation of numerical magnitude seems to be based on a mental mapping of numbers, oriented from left to right, the Mental Number Line (MNL; Dehaene, 2011), which assumes smaller numbers to be represented on the left side of visual space and larger numbers on its right side. Known as the Spatial Numerical Association of Response Codes (SNARC) (Dehaene et al., 1993), this effect facilitates responses given with the left hand when the stimulus is a smaller number, and with the right hand when the stimulus is a larger number. However, it has been pointed out that this effect can be culturally dependent, since right-to-left writers/readers show a “reverse” SNARC effect (Shaki et al., 2012; Shaki and Fischer, 2018).

The representation of affective valence has also been interpreted as an instance of left-right mapping, in this case of positive-negative affective valence, depending on the dominant hand. Because when we use our dominant hand, actions are performed more easily and smoothly compared to those performed by the non-dominant hand, a right-handed person would tend to attribute positive valence to stimuli placed on the right side (i.e., “Good Is right” mapping), whereas left-handers would show the opposite tendency (i.e., “Good Is Left” mapping). This is known as the body-specific hypothesis (Casasanto, 2009).

Moreover, with regards to the affective valence in the domain of brain asymmetries, the valence specific hypothesis (Wedding and Stalans, 1985; Davidson et al., 1990; Bassel and Schiff, 2001; Davidson, 2004; Najt et al., 2013) states that the LH (Left Hemisphere) is specialized for processing positive emotions, while the RH (Right Hemisphere) is specialized for processing negative emotions. Thus, positive stimuli (like happy faces) are recognized faster when shown in the respondents’ RVF (LH) than in their LVF (RH), whereas the recognition of negative stimuli (like sad faces) follows the opposite pattern. However, it is worth noticing that there is still a debate concerning the aforementioned theory, especially regarding the processing of positive emotions (e.g., Asthana and Manual, 2001).

Interestingly, a higher economic value can be cognitively processed as both more pleasant (because of its rewarding value) and as a larger numerical magnitude. For instance, a previous study, using a paradigm of lateralization, has shown that these two dimensions can be dissociable, suggesting that the attribution of affective valence may play a crucial role in price estimation (Giuliani et al., 2017).

The present study aims to extend this idea to banknotes (5€ and 100€) and advance our knowledge in the field of economic value perception. Indeed, excluding perceptual differences (like color, size, and luminance), which were held controlled in our experiment, a 100€ banknote represents both a larger numerical magnitude and a more rewarding (and thus more pleasant) stimulus than a 5€ banknote. Moreover, since different neurophysiological responses between money presented as a task-related reward (more salient) and a non-task-related reward (less salient; Zink et al., 2004), have been found, we focused on the latter. This allowed us to investigate the perception of money at a basic level.

As stated above, both numerical magnitude and affective valence can lead to laterality effects. Thus, in a lateralized recognition task, if the banknotes are coded with respect to their numerical magnitude, the recognition of 5€ (smaller magnitude) should be faster than 100€ (larger magnitude) in the LVF, whereas the opposite should be observed in the right visual RVF, following the principle of the MNL. However, if banknotes are coded with respect to their affective valence, the recognition of 100€ should be faster than 5€ in the RVF, because the former would be processed as a more pleasant (positive) stimulus than the latter. On the other hand, no differences should be expected in the LVF, which is the side where negative stimuli would be preferably mapped.

We expect our results to be in line with the latter pattern of laterality, in according to both valence hypothesis and body-specificity hypothesis. This result would indicate that money may be coded as an affective stimulus, rather than a numerical one. Moreover, this might suggest that we automatically attach an emotional value to money, regardless of its rewarding value within the task. From a theoretical perspective, this would suggest that a higher monetary value might be cognitively processed as a primary reward (Lea and Webley, 2006).

## Materials and Methods

### Participants

Forty-six participants (23 males and 23 females) with a mean age of 26.7 years (SE = 0.58) were recruited. All participants were right-handed students and native Italian speakers, with a mean hand preference index of 81.37 (SE = 2.24) as assessed by the Edinburgh Handedness Inventory (Oldfield, 1971).

The whole procedure was carried out in accordance with the principles of the Declaration of Helsinki, the protocol was approved by the Biomedical Research Ethics Committee, University of Chieti-Pescara, and participants gave written and informed consent before beginning the experiment.

### Stimuli and Procedure

We used the pictures of the 5€ and the 100€ banknotes as the experimental stimuli, and the scrambled versions of both as control stimuli. All the pictures were the same size (167 × 81 pixels; 5.09 × 2.30° of visual angle).

In a pretest, an independent sample of participants assessed the valence of both banknotes (see Supplementary Figure S1 for further details; Foroni et al., 2013 and Padulo et al., 2017 for the methodology).

**FIGURE 1:**
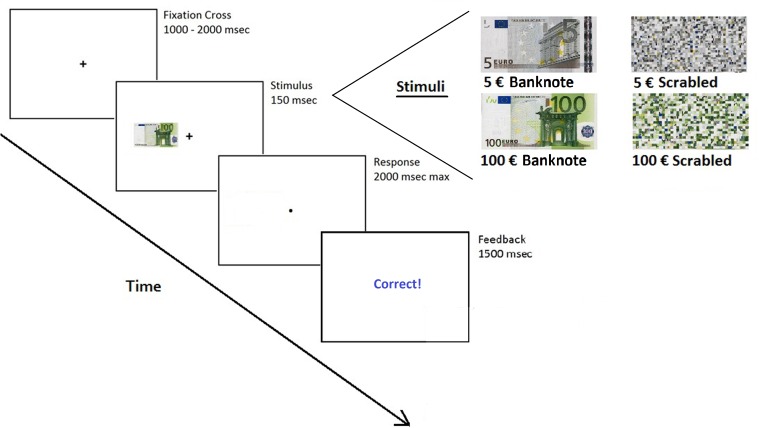
Experimental paradigm and stimuli. Each trial started with a fixation cross, after which one of the 4 stimuli (5€ banknote, 5€ scramble, 100€ banknote, 100€ scrambled) was shown for 150 ms. Participants had 2 s to identify (by a key press) whether the stimulus presented was a banknote or not. After the response, a feedback was given (Correct, Incorrect, Missed Response).

In the test session, participants were seated comfortably in the experimental room, in front of a computer monitor (15.6 inch, 1280 × 768 pixel) with the head located at a distance of approximately 50 cm. On-screen instructions were provided: “Each trial will be composed of a picture that will briefly appear either to the left or right of a fixation cross.” This picture could be a banknote or not a banknote. Press the key ‘p’ (right hand index) if the picture is a banknote or the key ‘u’ (left hand index) if it is not. Please focus on the fixation cross and provide your response as quickly (max 2 s) and accurately as you can”. Each trial started with a central fixation cross (1.6 × 1.6° of visual angle) presented for a random duration lasting between 1000 and 2000 ms. Subsequently, the stimulus was flashed for 150 ms at 7° eccentricity either to the left (LVF) or to the right (RVF) of the fixation cross. After the stimulus presentation, the screen went blank until the response was given (max 2000 ms). Feedback was provided for correct, incorrect and missed responses and then the next trial started. The identification keys (‘u’, ‘p’) were counterbalanced between participants. Each stimulus (4 in total) was presented 10 times in each of the two sides (for a total of 80 trials). The experiment was implemented using e-Prime 1.1 (Psychology Software Tools, Inc., Pittsburgh, PA, United States) and lasted about 2 min. See Figure 1 for procedure and stimuli.

## Results

Data analysis was carried out by means of the Statistica 8.0 software (StatSoft, Inc., Tulsa, OK, United States). Percentage of errors (PEs) and the mean reaction time of correct responses (RTs) from the stimulus onset until the response key press were collected. RTs +/- 3 SD were considered outliers and the PEs were then filtered by RTs (see Supplementary Table S1 for details on removed trials). Normality of our data was confirmed by the Kolmogorov-Smirnov test. Concerning PEs, the variable was not normally distributed and thus, to obtain a normal distribution, we applied a rank transformation (John et al., 2013) fractional as a percentage.

Then we performed an analysis of variance (ANOVA) with *post-hoc* Duncan’s comparisons at level of significance *p* = 0.05 for each of the two dependent variables (PEs, RTs), using the following within-subject factors: Visual Field (left, right), Stimulus Type (scrambled, banknote) and Value (5€, 100€). We also performed a second mixed-design ANOVA adding the arrangement of response keys as a between factor (Keys Arrangement; u-yes, p-yes) to the previous analysis.

**FIGURE 2:**
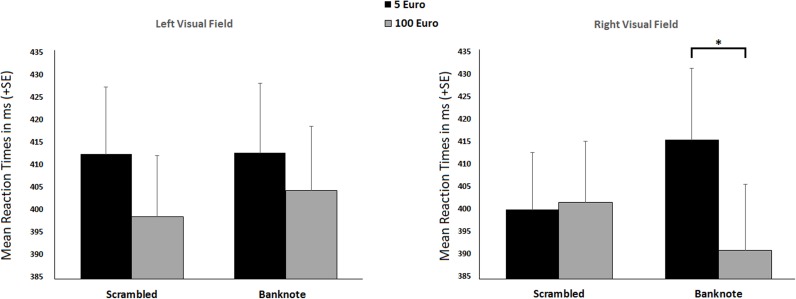
Mean response times (RTs) of recognition in the LVF (left) and RVF (right). The graphs refer to the significant three-way interaction: Visual Field × Stimulus Type × Value. Asterisk refers to significant *post-hoc* comparison.

Regarding the first analysis, RTs analysis showed a main effect of Value [*F*_(1,45)_ = 6.70; *p* = 0.013; $\eta_{p}^{2}$ = 0.13; 1 – β = 0.86] indicating that both the 100€ banknote and its scrambled version are recognized faster (mean = 398.99 ms; ES = 12.32) than both the 5€ banknote and its scrambled version (mean = 410.05; ES = 13.03). The three-way interaction, Visual Field × Stimulus Type × Value was also significant [*F*_(1,45)_ = 4.96; *p* = 0.031; $\eta_{p}^{2}$ = 0.10; 1 – β = 0.83; see Figure 2]. *Post-hoc* analysis indicated that, in the RVF, the 100€ banknote is recognized faster than the 5€ banknote (*p* = 0.004). No other significant differences were detected. PEs analysis showed no significant effects. Descriptive statistics are reported in Supplementary Table S2.

In the second analysis, the three-way interaction, Keys Arrangement × Visual Field × Stimulus Type was significant [*F*_(1,44)_ = 24.92; *p* < 0.001; $\eta_{p}^{2}$ = 0.36; 1 – β = 0.97). *Post-hoc* analysis indicated that in the u-yes (it is a banknote) condition, responses were faster when banknotes were shown in the LVF than in the RVF (*p* = 0.008). Conversely, in the p-yes condition, responses were faster when banknotes were shown in the RVF than in the LVF (*p* < 0.001). However, no Keys Arrangement × Stimulus Type × Value interaction was found [F(1,44) < 0.30; *p* > 0.59].

Datasets are available on request: the raw data supporting the conclusions of this manuscript will be made available by the authors, without undue reservation, to any qualified researcher.

## Discussion

Our results suggest that the cognitive processing underlying banknotes recognition may be primarily affective. The pilot experiment revealed that the 100€ banknote is judged as more positive than the 5€ banknote, which is in turn judged as neutral (neither negative nor positive). Thus, in line with the valence specific hypothesis (Wedding and Stalans, 1985; Davidson et al., 1990; Bassel and Schiff, 2001; Davidson, 2004; Najt et al., 2013), the faster recognition of 100€ (compared to 5€) in the RVF is probably due to the matching between the right side of visual space (primary processed by the LH) and positive valence. On the other hand, given the association between the left side of visual space (primary processed by the RH) and negative valence, no differential response between the neutral 5€ and positive 100€ were observed in the LVF. However, our results are also compatible with the body-specific hypothesis (Casasanto, 2009). In his seminal paper, Casasanto found that when both right and left-handed people have to choose on which side to assign subjectively “good” (or positive) stimuli, they would place them on their dominant side (right and left, respectively), while they do the opposite with “bad” (or negative) stimuli. The same tendency has been found with respect to positive and negative words (Kong, 2013), trading verbs (Vicario and Rumiati, 2015) and in an interpersonal choice task, where right-handers preferably chose faces of people shown on their right side, whereas left-handers preferred the opposite side (Zhao et al., 2016).

Our results also reveal additional details that contribute to uncovering the underlying cognitive processes involved in banknotes perception. First of all, the 100€ banknote is recognized faster (in the RVF) despite the fact that it is less common than the 5€ banknote, and possibly the more so for our sample of students (common stimuli are usually recognized faster than uncommon ones; see for instance Cuetos et al., 1999). This would suggest that the recognition based on valence may be more effective than that based on familiarity. However, since an effect of laterality for objects having different levels of familiarity has been shown in other species (but not in humans to the best of our knowledge), like dolphins (Blois-Heulin et al., 2012), this aspect would probably require further investigations.

A second consideration comes from the fact that banknotes recognition seems not to be influenced by the MNL, since 5€ (smaller magnitude) was not recognized faster than 100€ (larger magnitude) in the LVF, although 100€ was recognized faster than 5€ in the RVF. The lack of SNARC effect is also confirmed by the not significant interaction between the Keys arrangement and banknotes’ value, which would have been expected if numerical processing had been involved. However, a stimulus-response compatibility resembling the Simon Effect (Simon and Wolf, 1963; Hommel, 2011; D’Anselmo et al., 2015) was found. Responders were faster to select the proper response “yes, it is a banknote” when both 5€ and 100€ were shown to the side associated to that response (left in the group u-yes, and right in the group p-yes). These results suggest that while stimulus location was relevant in order to select the response, numerical magnitude was not. Thus, banknotes, although explicitly representing a numerical magnitude, may not necessary require access to numerical processing to be recognized. This leads us to infer that the affective state conveyed by a banknote is coded and mapped in the horizontal space before any other attribute of the stimulus.

The empirical evidence reported above, corroborates the idea that the basic perception of economic value might be primarily affective, rather than numerical, and it is automatically coded in relation to the horizontal space. However, future studies would need to take into consideration the participants’ socio-economic status and its potential interaction with the effects found here, which was not assessed in the present study.

From a theoretical perspective, our findings strengthen the idea that the economic value may be coded in respect of its affective valence and speaks in favor of the Drug Theory of money (Lea and Webley, 2006). This framework accounts for behavioral effects triggered by the perception of physical money, which may act like a primary reward, representing a need itself. Of note, money is also a tool, it is the means through which we satisfy our primary needs, such as eating, drinking, etc. Nevertheless, a monetary value, especially a higher one, may be intrinsically positive and rewarding just like a smiling face or a pleasant food. Subject to further investigation, our results could have practical implications. For example, if we consider that people are still largely using physical money, despite attempts by governments world-wide to switch to electronic payment systems, the following question may arise: could emotions related to the perception of physical money play a role in this phenomenon?

Although several other factors, both socio-cultural, and psychological, likely play a role in the aforementioned phenomenon, further investigation of the perception of money may help to clarify individuals’ attitude toward physical currencies.

## Author Contributions

FG and VM made substantial contributions to the conception or design of the work; critical revision for important intellectual content of the work; and acquisition, analysis, and interpretation of data for the work. FG drafted the work. AB, LT, and DP contributed to critical revision for important intellectual content of the work; agreement to be accountable for all aspects of the work in ensuring that questions related to the accuracy or integrity of any part of the work are appropriately investigated and resolved; and final approval of the version to be published.

## Conflict of Interest Statement

The authors declare that the research was conducted in the absence of any commercial or financial relationships that could be construed as a potential conflict of interest.
