# Supplementary material for: Side Biases in Euro Banknotes Recognition: The Horizontal Mapping of Monetary Value
Source: Front Psychol. 2018 Nov 21;9:2293. doi: 10.3389/fpsyg.2018.02293 (PMC6258740; doi:10.3389/fpsyg.2018.02293)
Supplement: Supplementary file 1 [file Table_1.DOCX]

Supplementary Material

**Side Biases in Euro Banknotes Recognition: The Horizontal Mapping of Monetary Value**

**Felice Giuliani^*^, Valerio Manippa, Alfredo Brancucci, Luca Tommasi & Davide Pietroni**

*** Felice Giuliani:** felice.giuliani@unich.it

# Supplementary Figures


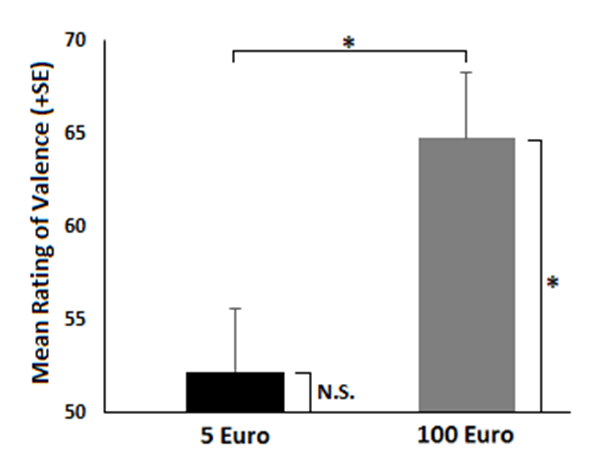


Supplementary figure S1.

A sample of 20 participants (9 males; 27.2 years, SE = 0.86) assessed the valence of both banknotes by using a 100-mm visual analogue scale (VAS). The question was: “How negative/positive is the item represented in the image?”; 0 = “very negative”, +100 = “very positive”. The 100€ banknote was assessed as more positive than the 5€ one (14.75 vs. 2.2; t = 3.15; p = .005) and it significantly differed from the neutrality point “50” (t = 4.19; p = < .001), whereas the 5€ did not. The diagram shows the mean Valence of 5€ and 100€ banknotes. The scale ranges from 0 (very negative) to 100 (very positive). 50 represents the center of the scale (neutral valence). Upper asterisk: significant difference between 5€ and 100€; lower asterisk: significant difference against 50 (neutral valence).
