# Supplementary material for: Side Biases in Euro Banknotes Recognition: The Horizontal Mapping of Monetary Value
Source: Front Psychol. 2018 Nov 21;9:2293. doi: 10.3389/fpsyg.2018.02293 (PMC6258740; doi:10.3389/fpsyg.2018.02293)
Supplement: Supplementary file 2 [file Table_2.DOCX]

Supplementary Material

**Side Biases in Euro Banknotes Recognition: The Horizontal Mapping of Monetary Value**

**Felice Giuliani^*^, Valerio Manippa, Alfredo Brancucci, Luca Tommasi & Davide Pietroni**

*** Felice Giuliani:** felice.giuliani@unich.it

# Supplementary Table 1. Removed trials. Number of outlier items removed from the analysis of correct trails for each condition (responses performed ± 3 SD from the mean RTs); total number of trials is in brackets.

| **Removed trials (total trials)** | | | | | | | | | | |  |
| --- | --- | --- | --- | --- | --- | --- | --- | --- | --- | --- | --- |
| **LVF** | | | | |  | **RVF** | | | | |  |
| **Scrambled** | |  | **Banknote** | |  | **Scrambled** | |  | **Banknote** | |  |
| **5€** | **100€** |  | **5€** | **100€** |  | **5€** | **100€** |  | **5€** | **100€** |  |
| 31 (430) | 34 (429) |  | 37 (424) | 32 (430) |  | 25 (435) | 41 (421) |  | 25 (435) | 40 (425) | |
|  |  |  |  |  |  |  |  |  |  |  | |
