# Supplementary material for: Side Biases in Euro Banknotes Recognition: The Horizontal Mapping of Monetary Value
Source: Front Psychol. 2018 Nov 21;9:2293. doi: 10.3389/fpsyg.2018.02293 (PMC6258740; doi:10.3389/fpsyg.2018.02293)
Supplement: Supplementary file 3 [file Table_3.DOCX]

Supplementary Material

**Side Biases in Euro Banknotes Recognition: The Horizontal Mapping of Monetary Value**

**Felice Giuliani^*^, Valerio Manippa, Alfredo Brancucci, Luca Tommasi & Davide Pietroni**

*** Felice Giuliani:** felice.giuliani@unich.it

# Supplementary Table 2. Descriptive results. Response times (RTs, ms) and mean errors (Errors, %) for each experimental condition; standard errors are in brackets. Data are reported separately for stimuli presented in the left and right visual field (LVF, RVF).

| **RTs (ms)** | | | | | | | | | | |
| --- | --- | --- | --- | --- | --- | --- | --- | --- | --- | --- |
| **LVF** | | | | |  | **RVF** | | | | |
| **Scrambled** | |  | **Banknote** | |  | **Scrambled** | |  | **Banknote** | |
| **5€** | **100€** |  | **5€** | **100€** |  | **5€** | **100€** |  | **5€** | **100€** |
| 412.31 | 398.62 |  | 412.52 | 404.33 |  | 400.10 | 401.69 |  | 415.29 | 391.30 |
| (14.62) | (13.30) |  | (15.21) | (14.00) |  | (12.49) | (13.32) |  | (15.57) | (14.31) |
|  |  |  |  |  |  |  |  |  |  |  |
| **Errors (rank %)** | | | | | | | | | | |
| **LVF** | | | | |  | **RVF** | | | | |
| **Scrambled** | |  | **Banknote** | |  | **Scrambled** | |  | **Banknote** | |
| **5€** | **100€** |  | **5€** | **100€** |  | **5€** | **100€** |  | **5€** | **100€** |
| 31.38 | 33.74 |  | 34.45 | 32.61 |  | 30.29 | 34.31 |  | 28.12 | 34.40 |
| (5.35) | (4.76) |  | (5.02) | (4.77) |  | (4.98) | (5.16) |  | (5.41) | (5.29) |
